# All-perovskite tandems go bifacial

**DOI:** 10.1038/s41377-022-01057-3

**Published:** 2023-01-03

**Authors:** Suhas Mahesh, Bin Chen, Edward H. Sargent

**Affiliations:** 1 The Edward S. Rogers Department of Electrical and Computer Engineering, University of Toronto, Toronto, ON M5S 3G4 Canada; 2 Department of Chemistry, Northwestern University, Evanston, IL 60208 USA; 3 Department of Electrical and Computer Engineering, Northwestern University, Evanston, IL 60208 USA

**Keywords:** Solar energy and photovoltaic technology, Photonic devices

## Abstract

All-perovskite tandem cells are attractive candidates for next-generation photovoltaic technology as they hold the potential to combine high-efficiency with low weight and reduced energy-payback times. Now, researchers show that such tandem cells can be engineering to be *bifacial*, allowing them to utilize stray light reflected off the surrounding environment, resulting in a 17% boost in the power output.

Continued progress in power conversion efficiency (PCE) for halide perovskite single-junction photovoltaic cells (currently 25.7%^1^) continues to draw intense interest. For the next generation of cells—those that will increase PCE materially over that achieved using even the most advanced single-junction silicon technology—tandem cells are of interest to go beyond the single-junction limit. High-efficiency cells (PCE > 30%) offer routes to further reductions in the levelized cost of electricity (LCOE), and halide perovskites, with their tunable bandgap, are excellent candidates for this approach.

Tandem solar cells use two absorbers instead of one, each specializing in a region of the solar spectrum, raising the theoretical efficiency to 46%^2^. This helps reduce the energy lost to electron-phonon scattering events (*thermalization*), the most prominent intrinsic loss mechanism in solar cells. The tandem concept has shown success in a number of architectures including perovskite-on-Si (31.25%^3^) and all-perovskite (27.4%^4^). All-perovskite tandems are particularly attractive as they hold the potential to combine high-efficiency with low weight and reduced energy-payback times.

Another architectural advance for increasing power generation is impacting the PV sector: bifaciality. Bifacial solar cells have increased current generation since they can absorb reflected and scattered light (*albedo*) that reaches the cell’s back-surface (Fig. 1). In typical solar cells, this surface is fully metalized and acts as a rear reflector. Bifacial PV replaces the metal with a transparent conductive oxide (TCO). Recent studies show that mounting modules on high albedo surfaces has the potential to increase energy yields by ~20–30%^5,6^. Bifacial c-Si modules are expected to increase market share considerably by 2028^7^.

**Fig. 1:**
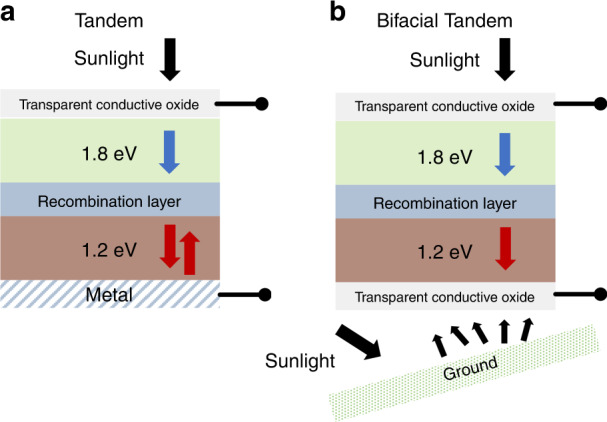
Architectures of the tandem and the Bifacial Tandem. **a** All-perovskite tandem. Light makes two passes through the structure after being reflected by the metal. **b** Bifacial all-perovskite tandem. Light reflected and scattered by the surroundings enters the rear cell through the TCO, increasing the generation.

Hairen Tan and co-workers have already made major contributions to perovskite tandem photovoltaics^4,8–13^. Writing in eLight, Tan and co-workers bring together the two previously described approaches and realize an impressive feat—the first bifacial all-perovskite tandem^14^. This is a p-i-n structure cell with perovskite absorbers of bandgaps 1.22 eV (FA_0.7_MA_0.3_Pb_0.5_Sn_0.5_I_3_) and 1.77 eV (FA_0.8_Cs_0.2_Pb(I_0.6_Br_0.4_)_3_). Their champion bifacial tandem exhibits an output power density of 28.51 mW/cm^2^, the equivalent of an efficiency of 28.51%, representing a 17% increase in power over the monofacial tandem.

There are complex optical and electronic phenomena to be dealt with in building bifacial devices. With the increased current density in the bottom cell, the bandgap of the top cell has to be narrowed to meet the current matching criterion, and layer thicknesses have to be re-optimized to account for the altered optics. The study varies the bandgap between 1.60 and 1.77 eV by changing the Br/I ratio in the perovskite, until current was found to be matched at 1.77 eV. TCO sputtering can cause interfacial degradation, lower the photoluminescence efficiency, and introduce s-kinks in the JV curve. To avoid this, the authors deployed a thin layer of SnO_2_ (20 nm) is deposited using Atomic Layer Deposition (ALD) to guard the transport layer and perovskite from sputtering damage due to ITO (80 nm). The success of this procedure is perhaps best demonstrated by the fact that the monofacial and bifacial tandems have near-identical open-circuit voltages. The study also performs optical modeling that shows that even with a moderate albedo of 34%, over 15% energy gain over monofacial tandems can be expected.

This work points the field of high-efficiency photovoltaics in a new direction, demonstrating the feasibility of realizing all-perovskite tandems in the bifacial configuration. Considering the rapidity with which bifacial modules are being adopted by the c-Si industry, it seems likely R&D related to bifaciality will increase in intensity in perovskite multijunction research as well.

Several aspects of bifacial all-perovskite tandems will benefit from continued investigation. Rigorous optical modeling is required to determine the bandgaps, layer thicknesses and tracking types that can maximize energy yield, while accounting for temporally changing albedo and spectral conditions. The rear C_60_/SnO_2_/ITO interfaces will benefit from structural and spectroscopic characterization to determine long-term failure modes. Scale up of such devices to the wafer scale will likely introduce fill-factor losses due to the series resistance introduced by the ITO. TCOs with the appropriate trade-off between transparency and sheet resistance have to be developed for large area bifacial tandem PV.
